# Radiomics in Medical Imaging: Methods, Applications, and Challenges

**DOI:** 10.3390/jimaging12060220

**Published:** 2026-05-23

**Authors:** Fnu Neha, Deepak Kumar Shukla

**Affiliations:** 1 Department of Computer Science, Kent State University, Kent, OH 44242, USA; 2 Rutgers Business School, Rutgers University, Newark, NJ 07102, USA; ds1640@scarletmail.rutgers.edu

**Keywords:** radiomics, artificial intelligence (AI), machine learning, medical image processing, automated diagnosis, digital health, computational radiology

## Abstract

Radiomics enables quantitative medical image analysis by converting imaging data into structured, high-dimensional feature representations for predictive modeling. Despite methodological developments and encouraging retrospective results, radiomics continue to face persistent challenges related to feature instability, limited reproducibility, validation bias, and restricted clinical translation. Existing reviews largely focus on application-specific outcomes or isolated pipeline components, with limited analysis of how interdependent design choices across acquisition, preprocessing, feature engineering, modeling, and evaluation collectively affect robustness and generalizability. This survey provides an end-to-end analysis of radiomics pipelines, examining how methodological decisions at each stage influence feature stability, model reliability, and translational validity. This paper reviews radiomic feature extraction, selection, and dimensionality reduction strategies; classical machine and deep learning–based modeling approaches; and ensemble and hybrid frameworks, with emphasis on validation protocols, data leakage prevention, and statistical reliability. Clinical applications are discussed with a focus on evaluation rigor rather than reported performance metrics. The survey identifies open challenges in standardization, domain shift, and clinical deployment, and outlines future directions such as hybrid radiomics–artificial intelligence models, multimodal fusion, federated learning, and standardized benchmarking.

## 1. Introduction

Medical imaging plays an important role in clinical decision-making, supporting diagnosis, prognosis, treatment planning, and disease monitoring. Advances in medical imaging have improved spatial resolution and increased availability across routine clinical workflows; however, clinical interpretation remains predominantly qualitative, subject to inter-observer variability and limited sensitivity to subtle phenotypic patterns embedded in high-dimensional image data [1]. These limitations highlight the need for quantitative image-derived biomarkers that complement visual interpretation and provide reproducible clinical evidence.

Radiomics addresses this need by enabling high-throughput extraction of quantitative descriptors from medical images, transforming visual information into structured, analyzable data [2]. Radiomic features characterize tissue morphology, intensity distributions, and spatial heterogeneity in a non-invasive manner and have been explored across diverse imaging modalities, including CT, MRI, PET, ultrasound, and digital pathology [3]. Through systematic quantification of imaging phenotypes, radiomics supports data-driven disease characterization and precision medicine approaches.

Radiomics originated from early quantitative texture analysis and computer-aided diagnosis studies developed to extract imaging biomarkers beyond visual interpretation. The term “radiomics” was introduced by Lambin et al. in 2012, emphasizing large-scale extraction of quantitative imaging features for precision medicine. Since then, radiomics has evolved from handcrafted feature analysis toward machine learning and deep learning–integrated frameworks with an increasing focus on reproducibility, harmonization, external validation, and clinical translation.

Radiomics has shown clinical relevance across multiple application domains, such as in oncology for tumor characterization, subtype differentiation, grading, outcome prediction, and therapy response assessment [4]. Applications have also extended to neurology and cardiology, where quantitative imaging phenotypes contribute to assessment of disease progression and functional outcomes beyond visually apparent patterns [5,6].

Despite growing interest, several methodological challenges, including variability in image acquisition, preprocessing, segmentation, and feature computation, limit the robustness and clinical translation of current radiomics research. High-dimensional feature spaces combined with limited cohort sizes increase the risk of overfitting and compromise reproducibility. Furthermore, external validation and multi-center evaluations remain insufficient, reducing confidence in model generalizability [7].

Existing radiomics surveys primarily provide component-wise descriptions of the workflow (e.g., feature extraction, modeling, or applications) [8,9,10,11,12,13]. However, they rarely analyze methodological dependencies, validation practices, or sources of variability systematically, leaving reproducibility and translational challenges insufficiently addressed. In contrast, this work adopts a dependency-aware and validation-centric perspective, analyzing how interdependent design choices across segmentation, feature engineering, and validation collectively influence reproducibility and generalization.

This survey adopts an end-to-end, methodology-centric analysis of radiomics pipelines, independent of application domain, modeling paradigm, or imaging modality. It analyzes how interdependent design choices across acquisition, preprocessing, segmentation, feature extraction, feature selection, modeling, and evaluation jointly determine feature stability, validation reliability, and clinical translatability. By linking pipeline decisions to sources of variability and reproducibility failure, this review establishes a unifying analytical framework that complements existing application-driven, deep learning–centric, and modality-specific surveys.

The main contributions of this paper are summarized as follows:

- A unified, dependency-aware overview of radiomics pipelines from image acquisition and preprocessing to feature extraction and predictive modeling.
- A comparative analysis of radiomic feature extraction, selection, dimensionality reduction, and modeling strategies, emphasizing their impact on robustness and reproducibility.
- A validation-centric review of major clinical application domains, prioritizing evaluation design over reported performance metrics.
- A critical discussion of open challenges and future research directions related to standardization, generalization, and clinical deployment.

The paper is organized as follows: Section 2 presents the research methodology. Section 3 presents the radiomics pipeline. Section 4 describes feature selection and dimensionality reduction. Section 5 discusses Validation Consideration. Section 6 discusses radiomics modeling approaches. Section 7 discusses the related work. Section 8 presents evaluation protocols and validation. Section 9 discusses challenges and limitations. Section 10 discusses the future work. Section 11 concludes the paper.

## 2. Research Methodology

This review adopts a reproducible methodology to identify, screen, and synthesize literature on radiomics, with emphasis on pipeline-level dependencies, feature stability, validation rigor, and clinical translatability. Unlike conventional surveys that summarize individual components, this review focuses on how methodological decisions across the radiomics pipeline collectively influence reproducibility and generalization. The process follows database-driven retrieval, multi-stage screening, and qualitative synthesis. Table 1 summarizes the key components of the methodology.

The literature retrieval process was conducted using combinations of keywords such as *radiomics*, *medical image feature extraction*, *quantitative imaging biomarkers*, *radiomics pipeline*, *feature stability*, *image harmonization*, and *radiomics validation*. The initial search yielded approximately 142 records across all databases. After duplicate removal and title/abstract screening, 72 studies were retained for full-text review. Following an eligibility assessment based on methodological relevance, validation rigor, and explicit treatment of feature-centric analysis, a final set of 70 studies was included for qualitative synthesis.

To ensure methodological completeness, the review additionally considers recent standardization efforts (e.g., IBSI guidelines), validation frameworks, and emerging hybrid radiomics approaches. Studies were not selected based on reported performance, but on their contribution to understanding sources of variability, reproducibility challenges, and methodological trade-offs across the radiomics pipeline.

Additionally, the synthesis was conducted using a methodology-centric comparative framework rather than performance-oriented aggregation. Selected studies were comparatively analyzed based on acquisition variability, preprocessing strategies, segmentation methods, feature engineering, feature stability, dimensionality reduction, validation design, reproducibility, and clinical translation considerations. Studies were grouped and interpreted according to methodological dependencies and validation-related factors instead of solely reported predictive performance metrics. This approach enabled the identification of recurring reproducibility challenges, validation gaps, and methodological limitations across radiomics pipelines.

### Research Question

This review is guided by the following research question (RQ), which frames radiomics as a pipeline-level representation and validation problem rather than a task-specific modeling approach:

- RQ1: How do design choices across the radiomics pipeline (acquisition, preprocessing, segmentation, feature extraction, and modeling) influence feature stability, reproducibility, and generalization?

## 3. Radiomics Pipeline

Radiomics follows a multi-stage pipeline that converts medical images into quantitative descriptors for predictive modeling. Each stage of this pipeline: (1) image acquisition; (2) segmentation; and (3) feature extraction and analysis, introduces methodological choices that directly influence feature stability, reproducibility, and downstream performance. It is important to understand these stages to interpret radiomics outcomes and evaluate their clinical reliability.

**Figure 1:**
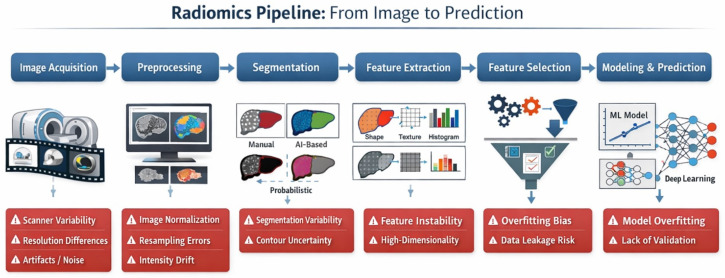
End-to-end radiomics pipeline with representative sources of variability and bias.

Figure 1 provides an end-to-end radiomics pipeline illustrating the progression from image acquisition to predictive modeling, with representative sources of variability and bias at each stage, including acquisition heterogeneity, preprocessing effects, segmentation uncertainty, feature instability, data leakage, and model overfitting.

### 3.1. Image Acquisition and Standardization

Image acquisition is a primary source of variability in radiomics pipelines [14,15]. Medical images are acquired using heterogeneous scanners and protocols, with variations in slice thickness, voxel spacing, reconstruction kernels, acquisition energy, and contrast timing. These factors directly influence image intensity distributions, spatial resolution, and noise characteristics, leading to systematic shifts in extracted radiomic features.

Inter-scanner variability and resolution differences impact texture and higher-order features, which are sensitive to interpolation, discretization, and noise statistics [16]. Inconsistent voxel anisotropy and resampling further impair feature stability and limit reproducibility across datasets, posing challenges for multi-center studies and external validation.

Standardization strategies aim to reduce acquisition-induced variability prior to feature extraction. Common approaches include:

1. **Spatial normalization**, such as resampling to isotropic voxel spacing using linear or spline interpolation to reduce resolution-induced effects [17].
2. **Intensity normalization**, including z-score normalization, histogram matching, Nyúl’s piecewise linear mapping, and modality-specific scaling (e.g., Hounsfield unit windowing in CT) [18].
3. **Gray-level discretization**, using fixed bin-width or fixed bin-count schemes to stabilize texture matrix computation [19].
4. **Feature-level harmonization**, such as ComBat, Bayesian ComBat, and deep harmonization variants, to remove scanner-related batch effects while preserving biologically relevant variation [20]. Recent work explores DL–based harmonization using GANs, and diffusion models to learn scanner-invariant representations. These approaches improve cross-site generalization but introduce additional training complexity and require multi-institutional data.

Acquisition variability affects radiomic feature reliability and stability, commonly quantified using intraclass correlation coefficients (ICC) [21]. Variations in scanner type, reconstruction kernel, and voxel resolution disproportionately reduce ICC values for texture and wavelet features, even under fixed segmentation. Shape features are comparatively robust, whereas higher-order features show the greatest sensitivity. Standardization and harmonization strategies mitigate these effects but introduce additional hyperparameters and modeling assumptions, requiring transparent reporting.

Table 2 summarizes key acquisition factors, affected feature categories, and mitigation strategies.

### 3.2. Region of Interest (ROI) Segmentation

Region of Interest (ROI) segmentation defines the spatial domain from which radiomic features are extracted. Accurate delineation of the ROI is critical, as feature values directly depend on the included voxels and boundary definition.

Segmentation can be performed manually, semi-automatically, or fully automatically. Manual delineation by expert annotators remains common in clinical studies; however, it is time-consuming and subject to inter- and intra-observer variability [22]. Semi-automatic methods reduce user effort through interactive refinement, while fully automatic approaches—primarily based on deep learning—enable scalable and reproducible segmentation.

The agreement between different segmentations is commonly quantified using overlap-based metrics. The Dice Similarity Coefficient (DSC) is defined as

(1) $$\mathrm{DSC}=\frac{2|A\cap B|}{\left|A|+|B\right|},$$

and the Jaccard Index (JI), or Intersection over Union (IoU), is given by

(2) $$\mathrm{IoU}=\frac{\left|A\cap B\right|}{\left|A\cup B\right|}=\frac{\left|A\cap B\right|}{\left|A|+|B|-|A\cap B\right|}.$$

The Jaccard Index provides a stricter measure of spatial agreement. Both metrics are monotonically related as

(3) $$\mathrm{DSC}=\frac{2\;\mathrm{IoU}}{1+\mathrm{IoU}},\;\mathrm{IoU}=\frac{\mathrm{DSC}}{2-\mathrm{DSC}}.$$

To address variability in manual segmentation, automated approaches based on DL—particularly U-Net and its variants—have become standard in medical image analysis [23]. These encoder–decoder architectures preserve spatial resolution through skip connections and are optimized using voxel-wise loss functions, enabling accurate delineation of complex anatomical structures when sufficient annotated data are available.

Beyond model development, several strategies have been proposed to reduce segmentation-induced variability. These include multi-observer consensus annotations, probabilistic segmentation frameworks, and perturbation-based robustness analysis. Emerging best practices suggest: (1) consistent inclusion or exclusion of lesion boundaries, as boundary voxels disproportionately influence feature values; (2) incorporation of peritumoral regions, which capture complementary biological signals such as tumor–host interaction; and (3) sensitivity analysis using controlled perturbations (e.g., dilation and erosion) to evaluate feature stability.

Despite these advances, segmentation uncertainty remains a major source of variability in radiomics. Even minor boundary perturbations can induce significant changes in both shape descriptors and higher-order texture features [24]. This sensitivity reflects the dependence of radiomic representations on precise voxel assignment and highlights an inherent trade-off between robustness and biological specificity.

**Figure 2:**
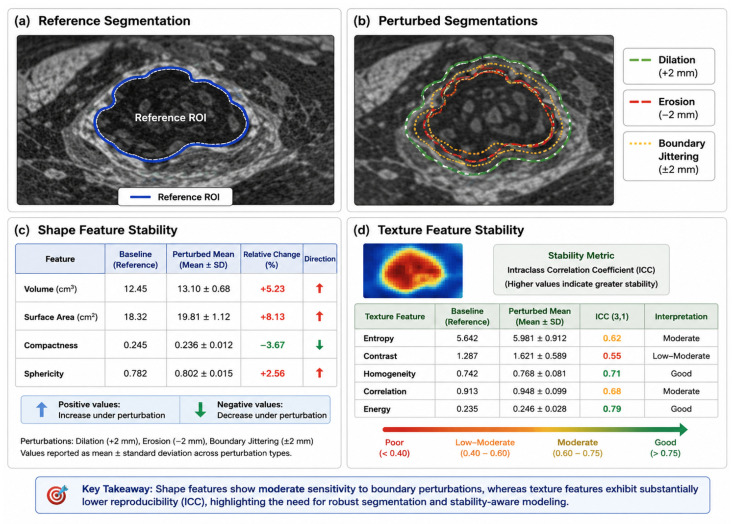
Quantitative assessment of segmentation-induced radiomic variability and feature stability. (**a**) Reference ROI segmentation used for baseline feature extraction. The white dashed contour indicates the original lesion boundary used as the reference segmentation. (**b**) Perturbed segmentations generated using controlled morphological operations, including dilation (+2 mm), erosion (−2 mm), and boundary jittering (±2 mm), to simulate segmentation uncertainty. The white dashed contour represents the baseline ROI boundary for comparison with perturbed segmentations. (**c**) Stability analysis of representative shape descriptors under segmentation perturbations, reported using relative percentage change between baseline and perturbed segmentations. Shape features demonstrate moderate sensitivity to ROI boundary modifications. (**d**) Texture feature reproducibility analysis using intraclass correlation coefficient (ICC)-based stability assessment. Texture descriptors exhibit comparatively lower reproducibility under perturbation due to voxel reassignment effects near lesion boundaries. Higher ICC values indicate greater feature stability and robustness.

Figure 2 quantitatively demonstrates the effect of segmentation perturbations on radiomic feature stability and reproducibility. Controlled boundary modifications, including dilation, erosion, and jittering, introduce measurable variability in both geometric and texture-based descriptors. Shape features exhibit moderate sensitivity to boundary alterations, whereas texture features demonstrate substantially lower reproducibility due to voxel reassignment near lesion margins. These observations highlight how segmentation uncertainty propagates through the radiomics pipeline and directly influences downstream model robustness, validation reliability, and clinical translatability.

### 3.3. Radiomic Feature Extraction

Radiomic feature extraction transforms segmented regions into quantitative descriptors that characterize underlying tissue phenotype [25]. These features are commonly grouped according to their mathematical formulation and the type of information they encode.

1. **First-order statistics** describe the statistical distribution of voxel intensities within the ROI, capturing measures of central tendency, dispersion, and intensity range without accounting for spatial relationships. The representative features include the mean intensity
  (4) $$\mu =\frac{1}{N}\sum_{i=1}^{N}x_{i},$$
  and variance
  (5) $$\sigma^{2}=\frac{1}{N}\sum_{i=1}^{N}{\left(x_{i}-\mu \right)}^{2},$$
  where $x_{i}$ denotes voxel intensities and *N* is the number of voxels.
2. **Shape features** quantify geometric properties of the segmented region, including volume, surface area, compactness, elongation, and sphericity. These descriptors are invariant to intensity scaling and capture morphological characteristics relevant to disease phenotype.
3. **Texture features** encode spatial relationships among voxel intensities and quantify intraregional heterogeneity. Common representations include gray-level co-occurrence matrices (GLCM), gray-level run-length matrices (GLRLM), gray-level size-zone matrices (GLSZM), neighboring gray-tone difference matrices (NGTDM), and gray-level dependence matrices (GLDM). For example, GLCM contrast is defined as
  (6) $$\sum_{i,j}{\left(i-j\right)}^{2}P\left(i,j\right),$$
  where P(i,j) denotes the joint probability of gray levels *i* and *j* at a specified spatial offset. Texture features exhibit moderate interpretability due to mathematical abstraction and lack of direct biological mapping.
4. **Higher-order features** are computed after applying image transformations such as wavelet decomposition, Laplacian-of-Gaussian filtering, Gabor filtering, or fractal analysis. These transformations enable multiscale and frequency-domain characterization by emphasizing structural patterns at different resolutions.

Table 3 provides a taxonomy of radiomic feature categories, highlighting their mathematical basis, stability, interpretability, and sensitivity. Stability refers to reproducibility under perturbations, while sensitivity reflects responsiveness to underlying biological variation. These are not redundant; highly sensitive features can be unstable under noise.

Standardization of radiomic features has been significantly advanced through initiatives such as the Image Biomarker Standardization Initiative (IBSI), including recent updates (IBSI v2), which define reproducible feature computation protocols across software frameworks, including PyRadiomics [25], Imaging Biomarker Explorer (IBEX) [26], MaZda [27], and the Computational Environment for Radiological Research (CERR) [28]. These efforts aim to reduce variability in higher-order features, including wavelet- and Laplacian-of-Gaussian–based descriptors, which are particularly sensitive to preprocessing parameters.

PyRadiomics is an open-source, Python-based library that provides Image Biomarker Standardisation Initiative-compliant feature extraction across first-order, shape, texture, and higher-order categories. IBEX is a MATLAB-based platform designed for quantitative imaging biomarker extraction, feature visualization, and sensitivity analysis in oncologic imaging. MaZda is a texture analysis software originally developed for medical image characterization, offering a broad range of statistical and model-based texture features. CERR is a MATLAB-based research framework developed for radiotherapy and imaging analysis, supporting radiomic feature extraction and multimodal data integration.

Table 4 compares commonly used radiomics software frameworks with respect to implementation environment, feature support, standardization compliance, and reproducibility considerations.

These tools support reproducibility, but variations in parameter settings, discretization strategies, and preprocessing configurations remain a significant source of inter-study variability. By far, PyRadiomics remains the most widely adopted framework due to IBSI compliance, reproducibility support, and integration with modern machine learning workflows.

To better connect pipeline design choices with reproducibility and clinical reliability, Table 5 summarizes the major methodological factors influencing feature stability, validation robustness, and translational generalization across radiomics workflows. The table emphasizes dependency-aware analysis rather than performance-oriented comparison alone.

Key methodological takeaway: Although texture and higher-order radiomic features provide increased sensitivity to tissue heterogeneity, they remain substantially more vulnerable to acquisition variability, preprocessing inconsistencies, and segmentation uncertainty than first-order or shape descriptors. Consequently, robust preprocessing standardization and reproducibility-aware feature analysis are essential for clinically reliable radiomics modeling.

## 4. Feature Selection and Dimensionality Reduction

Radiomics yields high-dimensional and highly correlated feature spaces from limited cohorts, leading to instability and reduced generalization. Feature selection and dimensionality reduction are therefore essential for robust and reproducible modeling.

### 4.1. Feature Selection Strategies

Feature selection aims to identify a subset of informative and non-redundant features that preserve discriminative power and reduce model complexity. Common feature selection techniques are categorized as filter, wrapper, and embedded methods.

1. **Filter methods** rank features independently of the predictive model using statistical relevance criteria. Representative techniques include variance thresholding, correlation analysis, and mutual information (MI) [29], defined as
  (7) $$MI\left(X;Y\right)=\sum_{x,y}p\left(x,y\right)\log\frac{p(x,y)}{p(x)p(y)},$$
  which quantifies the statistical dependence between a feature *X* and the target variable *Y*.
  A minimum redundancy–maximum relevance (mRMR) method extends this by prioritizing features that exhibit strong association with the outcome while reducing redundancy among selected features [29]. Filter methods offer computational efficiency and scalability in high-dimensional radiomics, but they do not explicitly account for feature interactions or model-specific behavior.
2. **Wrapper methods** integrate the learning algorithm into the selection process by iteratively evaluating feature subsets based on predictive performance [30]. Examples include recursive feature elimination (RFE), sequential forward selection, and genetic algorithms. These methods can capture complex feature interactions, but they incur higher computational costs and exhibit increased susceptibility to overfitting in small-sample radiomics studies.
3. **Embedded methods** perform feature selection during model training through regularization or intrinsic model constraints. Sparsity-inducing formulations, such as LASSO [31] and Elastic Net [32] optimize the objective function. This results in compact and interpretable feature subsets.
  Embedded methods offer a balance between interpretability and predictive performance, but their behavior remains influenced by feature scaling, correlation structure, and hyperparameter selection.

Feature selection in radiomics shows instability under data resampling. Selected feature subsets vary substantially across cross-validation folds or cohort splits. Low selection frequency reflects limited robustness and weak generalization. Post-hoc feature importance analyses should be interpreted cautiously, as importance scores are highly sensitive to feature correlation, scaling, and sampling variability and do not imply stable or causal relevance.

Key methodological takeaway: Stability-aware feature selection is critical in high-dimensional radiomics settings, as selected feature subsets frequently vary across cohort splits and validation folds. Methods that prioritize reproducibility and leakage-free evaluation are therefore essential for reliable model generalization.

### 4.2. Dimensionality Reduction

Dimensionality reduction complements explicit feature selection by projecting high-dimensional radiomic features into lower-dimensional latent representations.

Principal component analysis (PCA) is commonly employed to identify orthogonal components that capture maximal variance through eigen decomposition of the feature covariance matrix [33]. By retaining the leading components, PCA reduces redundancy and improves numerical stability, albeit at the expense of reduced feature interpretability.

Alternative approaches, including independent component analysis (ICA) [34], partial least squares (PLS) [35], and nonlinear manifold learning methods such as autoencoders [36] and kernel PCA [37], have been explored to capture higher-order dependencies and nonlinear structure in radiomics data. These methods increase representational compactness but introduce additional modeling complexity and stronger data requirements.

Other nonlinear dimensionality reduction methods include: t-Distributed Stochastic Neighbor Embedding (t-SNE) and Uniform Manifold Approximation and Projection (UMAP), which have gained widespread adoption for visualization and exploratory analysis of radiomics feature spaces. These methods preserve local or global structure in high-dimensional data but are primarily used for representation analysis rather than direct predictive modeling due to limited interpretability and reproducibility.

Feature selection and dimensionality reduction serve distinct but complementary roles. Feature selection identifies a subset of original features, while dimensionality reduction transforms features into latent components. Both are often applied sequentially to reduce redundancy and improve stability.

**Figure 3:**
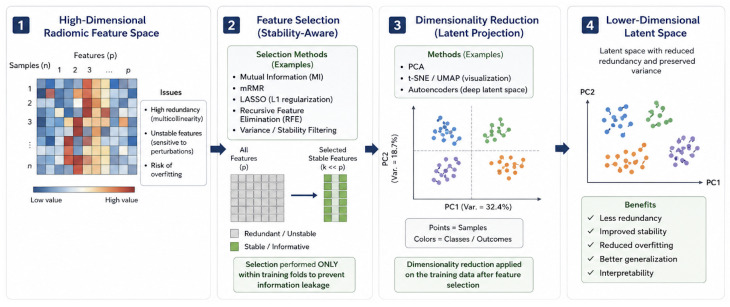
High-dimensional radiomic features are refined through stability-aware feature selection and latent-space projection to reduce redundancy, improve robustness, and enhance generalization. The ellipsis indicates omitted intermediate feature transformation and selection operations for visual simplicity.

Figure 3 presents a validation-aware workflow for feature selection and dimensionality reduction in radiomics. The pipeline begins with a high-dimensional radiomic feature space containing heterogeneous imaging descriptors extracted from multiple samples. These feature sets often exhibit redundancy, multicollinearity, instability under perturbations, and increased risk of overfitting. To address these limitations, stability-aware feature selection methods, including mutual information (MI), minimum redundancy maximum relevance (mRMR), LASSO, recursive feature elimination (RFE), and variance-based filtering, are applied to retain informative and reproducible features while eliminating unstable or redundant descriptors. The selected feature subset is subsequently transformed into a compact latent representation using dimensionality reduction techniques such as principal component analysis (PCA), t-SNE, UMAP, or autoencoders. The resulting lower-dimensional latent space preserves dominant variance patterns while improving stability, reducing overfitting, and enhancing generalization. Importantly, the workflow emphasizes leakage-free validation by ensuring that feature selection and dimensionality reduction are performed exclusively within training folds during cross-validation, thereby preventing information leakage and preserving unbiased model evaluation.

Key methodological takeaway: Feature selection and dimensionality reduction must be embedded exclusively within training folds during validation to prevent information leakage and artificially inflated performance estimates.

## 5. Validation Considerations

Feature selection and dimensionality reduction are closely coupled with the validation framework. Performing these steps outside the validation loop introduces information leakage and leads to optimistically biased performance estimates [38]. Embedding selection and reduction within cross-validation or nested validation schemes is therefore necessary for unbiased evaluation.

Leakage commonly arises when variance thresholding, mutual information ranking, LASSO regularization, or PCA are applied to the full dataset prior to data splitting. PCA offers partial interpretability as linear combinations of features, but lacks direct clinical meaning, hence, it is categorized as moderate-to-low interpretability. Such practices exploit test-set statistics during model construction, artificially inflating performance and yielding unstable feature subsets on independent evaluation. These effects are amplified in high-dimensional radiomics settings with limited sample sizes, directly undermining reproducibility, generalization, and translational reliability.

Table 6 summarizes common radiomics feature selection strategies and contrasts their robustness, leakage risk, and interpretability.

## 6. Radiomics Modeling Approaches

Radiomic features refined through selection or dimensionality reduction are incorporated into predictive models for clinical inference. Model choice directly affects accuracy, interpretability, robustness, and generalizability. Radiomics studies employ classical machine learning, deep learning–based, and hybrid modeling paradigms.

### 6.1. Classical Machine Learning (CML)-Based Radiomics

Classical machine learning (CML) algorithms remain widely adopted in radiomics due to their compatibility with handcrafted feature representations and effectiveness in limited-sample settings [39]. Commonly used models include support vector machines (SVM), logistic regression (LR), random forests (RF), and gradient-boosted (GB) trees (e.g., XGBoost and LightGBM). Additional approaches explored across applications include k-nearest neighbors (k-NN), naïve Bayes (NB) classifiers, decision trees (DT), and linear and quadratic discriminant analysis.

A key advantage of classical models lies in interpretability. Linear classifiers provide explicit decision functions and direct assessment of feature relevance. Tree-based ensembles offer feature importance measures that support qualitative model inspection. Data efficiency further contributes to their continued use in clinical radiomics studies.

At the same time, the performance of classical models remains tightly coupled to the stability and quality of handcrafted features. Sensitivity to feature redundancy, acquisition-induced variability, and preprocessing choices persists, while limited capacity to model complex, non-linear interactions constrains scalability as feature dimensionality increases.

**Figure 4:**
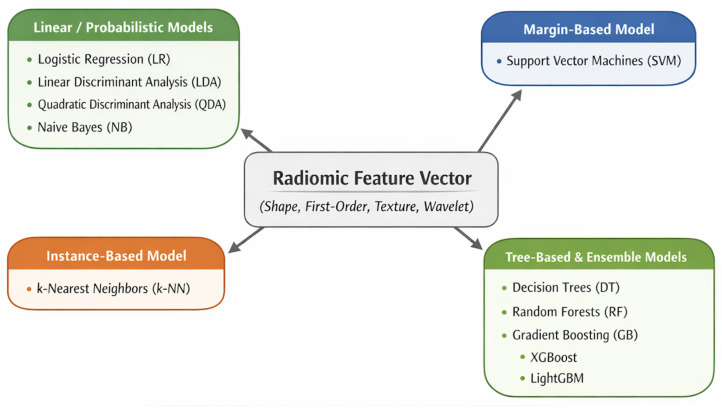
Classical Machine learning Models in Radiomics.

Figure 4 summarizes methodological trade-offs among commonly used classical machine learning models in radiomics. Linear models provide higher interpretability and reduced overfitting risk but may inadequately capture complex nonlinear relationships. Margin-based and ensemble methods often improve predictive performance in high-dimensional settings but exhibit increased sensitivity to feature instability, hyperparameter selection, and cohort variability. Consequently, model selection in radiomics should consider reproducibility, robustness, and validation reliability in addition to classification accuracy.

### 6.2. Deep Learning (DL)–Based Radiomics

Deep learning (DL)–based radiomics replaces handcrafted feature pipelines with end-to-end representation learning directly from imaging data [39]. Convolutional neural networks (CNNs) learn hierarchical, spatially structured features from raw or minimally processed images [40]. CNNs have shown strong performance in lesion detection, tumor classification, grading, and outcome prediction.

DL-based approaches enable modeling of complex imaging patterns that are difficult to capture using predefined descriptors and have shown promise across CT, MRI, PET, and digital pathology. However, their effectiveness is strongly dependent on data volume, annotation quality, and cohort diversity. Limited sample sizes and homogeneous datasets increase susceptibility to overfitting and restrict generalization. In addition, architectural variability, training stochasticity, and limited interpretability complicate reproducibility and clinical acceptance.

### 6.3. Ensemble and Hybrid Radiomics Frameworks

Ensemble and hybrid radiomics frameworks integrate multiple learners, feature spaces, or data modalities to mitigate limitations of individual models and improve robustness and generalization.

Boosting-based ensembles, including AdaBoost and GB, construct additive models by sequentially weighting weak learners and show strong performance when radiomic feature sets contain informative yet noisy descriptors [41]. Sensitivity to feature instability and preprocessing variability, however, remains a limiting factor. Bagging-based approaches, such as RFs and random subspace methods, reduce variance through aggregation of decorrelated learners.

Stacking (stacked generalization) combines heterogeneous base learners—such as SVMs, RFs, and LR—via a meta-model trained on their predictive outputs [42]. Related formulations, including blending, Bayesian model averaging, and super learner frameworks, have also been explored to integrate complementary decision functions. In radiomics, stacking-based ensembles have shown improved robustness in heterogeneous datasets, provided strict separation between training and validation folds is maintained.

Fusion-based hybrid frameworks integrate radiomic features with DL representations, clinical variables, or multi-modal imaging data [43,44]. Feature-level fusion aggregates heterogeneous descriptors prior to modeling, whereas decision-level fusion combines predictions from independently trained models. Intermediate fusion strategies, including attention-based integration, graph-based modeling, and multi-task learning, have been analyzed to capture cross-modal dependencies.

Despite increased flexibility, ensemble and hybrid frameworks introduce additional complexity through multi-stage training and expanded hyperparameter spaces. This complexity challenges reproducibility, deployment, and clinical integration, underscoring the need for rigorous validation and transparent reporting.

### 6.4. Radiomics vs. Deep Learning

Radiomics and DL represent distinct and complementary approaches to quantitative medical image analysis. Radiomics relies on handcrafted, mathematically defined features combined with conventional ML models, whereas DL derives hierarchical feature representations directly from imaging data. These methodological differences have direct implications for data requirements, interpretability, robustness, validation complexity, and clinical translation.

Radiomics is well-suited to limited-cohort studies and supports transparent, feature-level interpretation aligned with established imaging biomarkers. In contrast, DL excels in large-scale datasets and complex perceptual tasks but introduces challenges related to explainability, reproducibility, and deployment. Consequently, neither paradigm is universally optimal across clinical scenarios.

**Figure 5:**
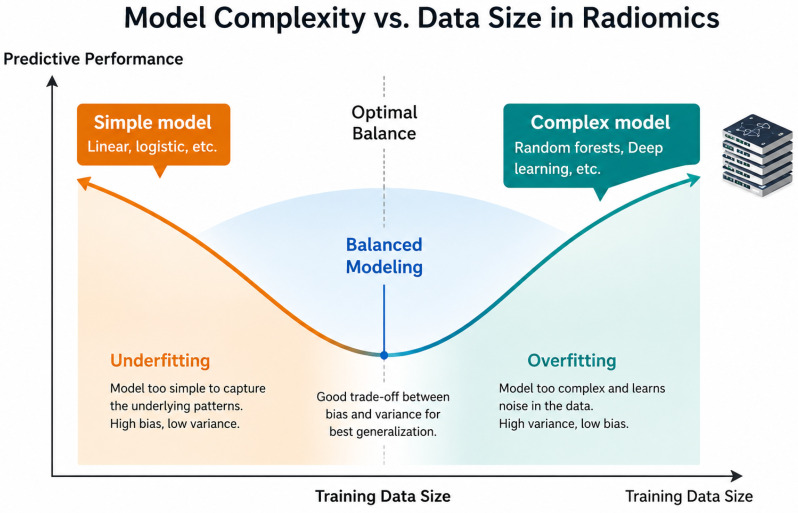
Model complexity vs. data size trade-off in radiomics.

Figure 5 illustrates the trade-off between model complexity and training data size in radiomics, highlighting underfitting, overfitting, and the setting for optimal generalization. Predictive performance depends on matching model capacity to available data, as simple models underfit high-dimensional feature spaces, whereas complex models overfit when data are limited.

In Figure 5 *Optimal balance* refers to the point where model capacity matches data availability, maximizing generalization. *Balanced modeling* denotes the regime around this point where neither underfitting nor overfitting dominates.

Table 7 summarizes key methodological and practical distinctions between radiomics and DL approaches.

The growing adoption of hybrid radiomics frameworks reflects that radiomics and DL address complementary problems of clinical imaging analysis. Therefore, a comparative understanding of these frameworks is important for selecting appropriate modeling strategies and designing robust, clinically translatable studies.

## 7. Related Work

Radiomics enables the extraction of quantitative imaging biomarkers that support disease characterization, prognostic assessment, and evaluation of therapeutic response. Features derived from baseline imaging have been associated with clinical outcomes, and longitudinal analysis captures temporal changes related to disease progression or treatment response. Across CT and MRI, radiomic analysis has been applied to nephrology, neurological, cardiovascular, pulmonary, and musculoskeletal conditions, facilitating objective assessment of structural and tissue-level alterations.

Applications include outcome prediction in neurodegenerative disease and stroke, risk stratification in cardiovascular disorders using coronary CT angiography and cardiac MRI [45], characterization of parenchymal abnormalities in pulmonary disease, and quantitative evaluation of bone and cartilage degeneration in musculoskeletal imaging [46].

Yu et al. developed a CT-based radiomics and ML approach using histogram, texture, and gradient features combined with a linear SVM to differentiate renal tumor subtypes and oncocytoma [47]. Lu et al. developed a multimodal MRI-based radiomics framework using intensity, texture, and shape features combined with hierarchical ML classifiers (primarily SVMs) to infer key molecular characteristics of gliomas and enable noninvasive stratification according to WHO-defined molecular subtypes [48]. Feng et al. proposed an SVM with RFE model, combined with a Synthetic Minority Oversampling Technique (SMOTE), for the quantitative texture analysis of CT-images, to differentiate between different types of renal masses [49]. SMOTE generates synthetic minority-class samples by interpolating between nearest-neighbor instances to address class imbalance during model training [50].

Chaddad et al. proposed a deep radiomics framework for survival prediction in recurrent glioblastoma by extracting CNN-based deep radiomic features from MRI and using RF to stratify patients into survival risk groups, showing the prognostic advantage of deep features over handcrafted descriptors [51]. Li et al. developed a deep CNN–based survival prediction framework for rectal cancer using PET/CT imaging, incorporating spatial pyramid pooling to accommodate variable tumor sizes [52]. Chen et al. developed a radiomics-based ML framework using texture features extracted from MRI and multiple feature-selection–classifier combinations, showing that distance correlation–based feature selection combined with linear discriminant analysis (LDA) or LR can differentiate glioblastoma from metastatic brain tumors [53].

Yi et al. applied SVM and RFs, with radiomic features, to differentiate between low-grade and high-grade clear cell renal cell carcinoma (ccRCC)—a subtype of RCC [54]. Gitto et al. developed a radiomics–based ML model using first-order and texture features extracted from MRI and an AdaBoost ensemble classifier to differentiate low-grade from high-grade cartilaginous bone tumors [55]. Deng et al. applied CT texture analysis using a filtration–histogram method with various spatial scaling filters to derive features that capture heterogeneity [56]. Various statistical metrics such as Entropy, kurtosis, skewness, and mean positive pixel features were extracted from CT images, with ROI drawn manually on the largest tumor cross-section. The model was assessed using LR analysis to segregate benign and malignant tumors. Erdim et al. employed DT, k-NN, LR, SVM, NB, RF, Feed Forward Neural Network (FFN), and locally weighted learning, to separate benign from malignant renal tumors using texture features of CT scans [57]. Their feature-selection strategy utilizes a greedy search for optimizing the feature set. Pie et al. proposed statistical analysis of a radiomics nomogram that incorporates a radiomics signature and clinical factors for the preoperative differentiation between fat deposits and ccRCC [58].

Sun et al. proposed an SVM-based approach, combining qualitative radiologic features with quantitative texture features, to differentiate benign from malignant renal tumors using CT-images [59]. Uhlig et al. developed a standalone XGBoost classifier and LR to classify five renal tumor subtypes [60]. Both techniques use RFE to prune undesired features.

Wang et al. applied RF, SVM, and LR from CT images to differentiate ccRCC from non-ccRCC [61]. Correlation analysis removed redundant features. LR identified key predictors: variance, High Gray Level Run Emphasis (HGLRE), and minimum intensity. Gurbani et al. evaluated a CT-based radiomics and ML framework for identifying aggressive tumor features in RCC, focusing on high nuclear grade and sarcomatoid differentiation in large RCCs using non-contrast and portal venous phase CT images [62]. Volumetric radiomic features were extracted from 3D tumor segmentations, and multiple ML classifiers (XGBoost, RF, and SVM) were investigated with feature ranking and selection strategies.

Alhussaini et al. applied RF, SVM, KNN, LR, and NB to differentiate malignant tumors using limited handcrafted radiomics features extracted from CT scans [63]. Features are reduced using sparsity-driven regularization that eliminates less informative variables. The filtered features are classified using ML techniques. Lam et al. developed an MRI-based radiomics model using handcrafted features and a LightGBM classifier to predict tumor mutational burden in lower-grade gliomas [64].

He et al. proposed an ensemble framework for malignancy risk prediction in cystic renal lesions using CT-scans [65]. The method integrates handcrafted radiomics features with DL-features extracted from a pretrained residual network. GB, XGBoost, and DT are employed to combine the derived features for the classification. Kumar et al. evaluated MRI radiomic features with five ML classifiers (SVM, RF, GB, NB, and AdaBoost) for low- vs. high-grade glioma classification [66]. Xu et al. integrated radiomics on CT-scans with clinical attributes (demographics, vital signs, and comorbidities) and ML (RF and XGBoost) and CNN for binary classification of renal tumors [67].

Magnuska et al. integrated radiomics and DL-based features for ultrasound (US)-based binary breast-tumor classification [68]. The model employs SVM, RF and LR for tumor categorization.

Chaddad et al. developed an MRI-based radiomics framework using XGBoost and RF with feature selection to differentiate lower-grade gliomas from glioblastoma and to derive radiomic risk signatures from pre-treatment scans for tumor classification and survival prediction [69]. Kilicarslan et al. proposed an ensemble deep learning framework for RCC subtype classification using MRI data [70]. Features are extracted via transfer learning using pretrained DenseNet architectures, followed by Global Average Pooling (GAP) to aggregate spatial activations into compact representations. The resulting feature vectors are concatenated and classified using SVM.

Multiple CT-based radiomics studies have studied tumor characterization using handcrafted texture, histogram, and shape descriptors combined with conventional machine learning classifiers such as SVM, RF, LR, and XGBoost. Across these studies, feature instability, limited cohort diversity, manual ROI delineation, and insufficient external validation remained recurring methodological limitations despite encouraging classification performance.

Table 8 and Table 9 present a comparison of classical radiomics and ML-based studies.

Table 10 presents a comparison of deep radiomics and hybrid radiomics–DL frameworks.

## 8. Evaluation Protocols and Validation

Evaluation protocols determine the reliability, generalization, and translational potential of radiomics models. Given the high dimensionality of radiomic feature spaces and the limited size of most imaging cohorts, rigorous validation is required to avoid biased performance estimates. Robust evaluation requires strict control of train/test separation, principled resampling strategies, external validation, and comprehensive performance assessment.

### 8.1. Train/Test Leakage and Cross-Validation

Train/test leakage occurs when information from evaluation data influences model development. Data leakage commonly arises when preprocessing steps such as normalization, harmonization, feature selection, or dimensionality reduction are performed before cross-validation [71]. In radiomics, the high feature-to-sample ratio amplifies the impact of such leakage, leading to inflated performance estimates.

Let $D={\left\{\left(x_{i},y_{i}\right)\right\}}_{i=1}^{N}$ denote a dataset. In *k*-fold cross-validation, *D* is partitioned into disjoint subsets $\left\{D_{1},\ldots ,D_{k}\right\}$. For each fold *j*, a model is trained on $D\setminus D_{j}$ and evaluated on $D_{j}$. The estimated performance metric $\hat{M}$ is

(8) $$\hat{M}=\frac{1}{k}\sum_{j=1}^{k}M\left(D_{j}\right),$$

where M(·) denotes the evaluation metric.

Hyperparameter tuning, feature selection, and dimensionality reduction must be restricted to the training portion of each fold. Nested cross-validation enforces this constraint by introducing an inner loop for model selection and an outer loop for unbiased performance estimation.

Bootstrapping is employed to estimate performance variability. Given *B* bootstrap samples drawn with replacement from *D*, confidence intervals can be computed from the empirical distribution of the performance metric.

Given *B* bootstrap resamples, the (1−α) confidence interval for a performance metric *M* is estimated as

(9) $${\mathrm{CI}}_{1-\alpha }=\left[M^{\left(\alpha /2\right)},\;M^{\left(1-\alpha /2\right)}\right],$$

where $M^{\left(q\right)}$ denotes the *q*-quantile of the empirical bootstrap distribution of *M*.

### 8.2. External and Temporal Validation

External validation evaluates a trained model on an independent dataset collected under different acquisition conditions, scanners, or institutions. This setting tests robustness to distributional shift, which is common in radiomics due to scanner heterogeneity and protocol variability.

Temporal validation represents a related strategy in which models are trained on earlier cases and evaluated on data acquired at a later time point. This approach assesses stability under evolving clinical practice and acquisition settings.

Despite their importance, external and temporal validation remain underrepresented in radiomics studies. Reported performance frequently declines under independent testing, indicating sensitivity to cohort composition and acquisition variability.

Futhermore, data leakage remains a documented issue in radiomics, particularly in small-cohort studies where preprocessing and feature selection are incorrectly applied prior to cross-validation [9,72]. Several studies have reported performance degradation under external validation, indicating inflated results due to improper validation protocols.

**Figure 6:**
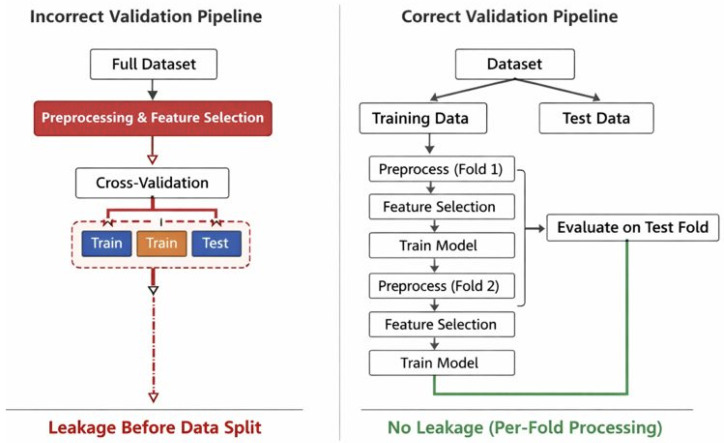
Comparison of incorrect and correct radiomics validation pipelines. (**Left**) preprocessing and feature selection applied before data splitting introduce leakage. (**Right**) preprocessing and feature selection are performed independently within each training fold. Models trained per fold are evaluated on the corresponding held-out fold (green path).

Figure 6 illustrates incorrect versus correct validation pipelines in radiomics. The left panel illustrates a common data leakage scenario in which preprocessing and feature selection are performed on the full dataset prior to cross-validation, resulting in optimistically biased performance estimates. The right panel depicts a leakage-free evaluation protocol, where data splitting precedes all preprocessing and feature selection steps, which are executed independently within each training fold before evaluation on held-out data. The brackets indicate fold-specific preprocessing steps, not shared operations. Each fold maintains strict separation between training and testing data.

### 8.3. Performance Metrics

Accuracy is insufficient for evaluating radiomics models, particularly in imbalanced datasets. Discriminative ability is commonly assessed using the area under the receiver operating characteristic curve (AUC), which evaluates ranking performance independent of decision thresholds [73].

For binary classification: sensitivity, specificity, precision and F1-score are reported to account for class imbalance.

Calibration assesses agreement between predicted probabilities ${\hat{p}}_{i}$ and observed outcomes $y_{i}$ [74]. Brier score provides a quantitative measure of probabilistic accuracy [75].

(10) $$\mathrm{Brier}=\frac{1}{N}\sum_{i=1}^{N}{\left({\hat{p}}_{i}-y_{i}\right)}^{2}.$$

Calibration curves and goodness-of-fit tests further characterize systematic deviations between predicted and observed risks. Statistical comparison of AUCs can be performed using DeLong’s test in paired evaluation settings.

In imbalanced datasets, the area under the precision–recall curve (AUPRC) provides a more informative evaluation than AUC, as it emphasizes performance on the minority class.

### 8.4. Stability and Statistical Significance Analysis

Feature and model stability are critical considerations in radiomics. Stability is commonly assessed using test–retest analysis or perturbation-based resampling and quantified via the intraclass correlation coefficient (ICC):

(11) $$\mathrm{ICC}=\frac{\sigma_{\mathrm{between}}^{2}}{\sigma_{\mathrm{between}}^{2}+\sigma_{\mathrm{within}}^{2}}.$$

ICC measures feature reproducibility across repeated measurements.

Features or models with low ICC values reflect sensitivity to acquisition or segmentation variability and should be excluded to improve reproducibility [21]. Permutation testing is used to assess the statistical significance of model performance by comparing observed results against a null distribution generated through random label permutations, thereby guarding against spurious associations in high-dimensional feature spaces.

ICC < 0.5 indicates poor reliability, 0.5–0.75 moderate, 0.75–0.9 good, and >0.9 excellent stability. Low ICC features should be excluded or stabilized using harmonization and robust feature selection strategies.

### 8.5. Radiomics Quality Score (RQS)

To improve methodological rigor and reproducibility in radiomics, Lambin et al. introduced the Radiomics Quality Score (RQS), a 36-point framework for assessing study quality, validation rigor, feature robustness, and clinical utility [76]. The RQS emphasizes imaging standardization, reproducibility analysis, feature reduction, external validation, and transparent reporting practices. Due to increasing variability in radiomics methodologies, RQS has become an important tool for evaluating the reliability and translational potential of radiomics studies.

## 9. Challenges and Limitations

**Figure 7:**
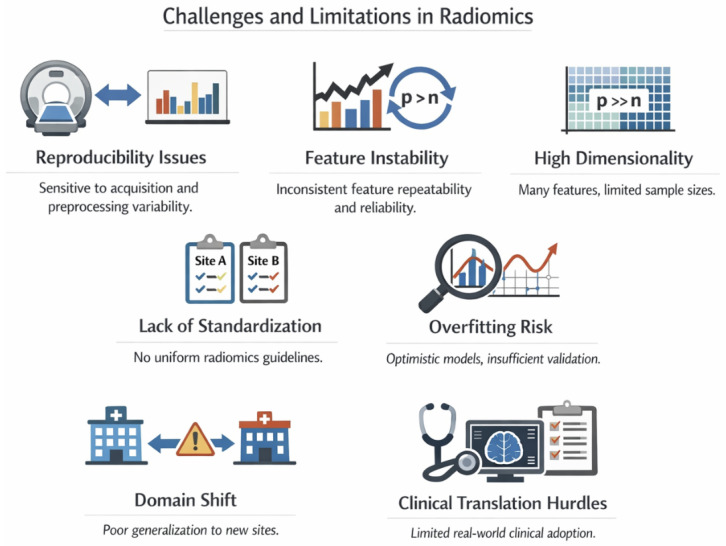
Overview of key challenges and limitations in radiomics.

Despite sustained methodological progress, radiomics continues to face fundamental limitations that impede reproducibility, generalization, and clinical translation, as shown in Figure 7. These challenges arise from the sensitivity of handcrafted features to acquisition and preprocessing variability, intrinsic statistical constraints of high-dimensional modeling under limited data, and persistent weaknesses in validation and standardization practices.

### 9.1. Reproducibility and Pipeline Sensitivity

Radiomic features show strong dependence on imaging acquisition parameters, reconstruction algorithms, preprocessing choices, and segmentation strategies. Even minor variations in scanner settings, voxel resolution, intensity discretization, or region-of-interest delineation can substantially alter feature distributions, with higher-order texture features being particularly affected [77,78]. This sensitivity produces pronounced inter-site variability and undermines robustness under domain shift, leading to degraded performance when models trained on single-center data are evaluated on external cohorts.

### 9.2. Feature Stability and Reliability

Radiomic features present heterogeneous stability under test–retest conditions and controlled perturbations. First-order intensity statistics and shape descriptors generally show higher repeatability, whereas texture- and filter-based features are highly sensitive to noise, quantization, and spatial resolution. Unstable features compromise biomarker interpretability and model reliability, particularly when feature selection prioritizes discriminative power without accounting for robustness. Inconsistent reporting of stability analyses further limits reproducibility and cross-study comparison. Despite existing mitigation strategies, feature instability remains an unresolved problem. Harmonization reduces scanner effects but may remove biologically relevant variation, while robust feature selection improves stability at the cost of reduced feature diversity [79].

### 9.3. High Dimensionality and Limited Sample Sizes

Radiomics typically operates in high-dimensional settings (p≫n), where hundreds to thousands of features are extracted from relatively small patient cohorts. This imbalance results in variance inflation, unstable parameter estimates, and increased risk of spurious associations [80]. Although feature selection and regularization mitigate dimensionality, they do not overcome fundamental constraints related to identifiability, statistical power, and uncertainty estimation. These issues are amplified in multi-class classification and survival analysis, where reliable stratified validation and subgroup analysis are often infeasible.

### 9.4. Lack of Standardization Across Studies

The absence of end-to-end standardization across radiomics pipelines remains a major barrier to reproducibility. Variability in acquisition protocols, preprocessing workflows, feature definitions, discretization schemes, software implementations, and validation designs leads to inconsistent feature representations and non-comparable results. While standardized feature sets and reporting guidelines have been proposed, their adoption remains uneven, limiting cumulative evidence synthesis and robust meta-analytic evaluation.

Despite strong predictive performance reported in many radiomics studies, clinical interpretability remains a major challenge. Numerous studies primarily report aggregate model metrics such as Area Under Curve, accuracy, and *p*-values without adequately describing the specific radiomic features contributing to model predictions. For clinical adoption, especially in radiology, it is important to report and interpret the biologically or visually relevant features associated with the study objective. Transparent explanation of significant radiomic descriptors may improve trust, reproducibility, and communication between computational researchers and radiologists. Consequently, future radiomics studies should emphasize feature-level interpretability alongside statistical model performance.

### 9.5. Overfitting and Validation Bias

Overfitting is pervasive in radiomics due to high feature dimensionality, extensive model and hyperparameter exploration, and insufficiently rigorous validation strategies. Feature selection and tuning performed outside nested validation frameworks introduce optimistic bias, inflating reported performance. Increased model complexity through ensemble or hybrid approaches further exacerbates this issue under data-limited conditions. Performance metrics are often reported without adequate uncertainty quantification, obscuring true generalization capability.

### 9.6. Barriers to Clinical Translation

Despite promising retrospective findings, clinical adoption of radiomics remains limited by challenges related to reproducibility, workflow integration, and validation reliability. Many studies lack prospective or longitudinal evaluation and provide insufficient evidence of real-world clinical utility. Translation is further constrained by dependence on accurate segmentation, complex preprocessing pipelines, limited interpretability, inadequate uncertainty modeling, and sensitivity to acquisition variability. In addition, deployment within hospital imaging ecosystems requires interoperability with Picture Archiving and Communication Systems (PACS), standardized acquisition protocols, transparent multicenter validation, regulatory compliance, and data privacy safeguards. Consequently, successful clinical translation depends not only on predictive performance, but also on robustness, reproducibility, and seamless integration into clinical workflows.

Overall, radiomics pipelines remain fragile to acquisition and preprocessing variability, statistically constrained by high-dimensional modeling under limited data, and predominantly evaluated using retrospective designs. While methodological advances have increased feature complexity and model capacity, they have not resolved core issues related to stability, standardization, and generalization, resulting in performance degradation under independent validation and clinical deployment.

## 10. Discussion and Future Work

Radiomics has shown sustained relevance in quantitative medical image analysis, however, its future impact depends on methodological refinement, integration with emerging learning models, and alignment with clinical practice. This section outlines key directions that build on established strengths while addressing persistent limitations.

### 10.1. Interpretability and Feature Relevance

Radiomics offers explicit feature definitions, enabling feature-level interpretability not inherently available in end-to-end DL models. Feature relevance can be examined through coefficient analysis, permutation importance, and stability metrics under resampling or perturbation [81]. These analyses support traceability of model decisions to specific image-derived properties.

However, statistical relevance derived from model optimization does not guarantee clinical relevance. Feature importance rankings are sensitive to correlation structure, regularization strength, and sampling variability. Future radiomics studies should distinguish predictive contribution from feature robustness by jointly reporting effect size, selection frequency across resampling, and test–retest stability. Feature relevance analysis that ignores stability constraints risks promoting non-reproducible biomarkers. Additionally, future work should focus on mapping radiomic features to clinically meaningful phenotypes using explainable models, integrating radiomics with pathology and genomics, and developing standardized feature ontologies to bridge the gap between mathematical descriptors and biological interpretation.

### 10.2. Hybrid Radiomics–DL and Transformer-Based Models

Hybrid frameworks combine handcrafted radiomic features with learned representations to extract complementary information. DL components operate either on image patches or feature embeddings, while radiomics provides structured, low-dimensional descriptors. Transformer-based architectures extend this framework by modeling feature interactions through self-attention mechanisms rather than fixed convolutional locality.

In hybrid radiomics–transformer models, radiomic features can be treated as tokens, enabling attention-based weighting and interaction modeling. This allows adaptive feature relevance estimation and long-range dependency modeling. Technical challenges include feature scaling compatibility, attention collapse in low samples, and increased variance due to model capacity. These models require strict regularization and external validation to avoid capacity-driven overfitting.

### 10.3. Multimodal Fusion

Radiomics supports multimodal fusion through its compatibility with heterogeneous data types. Fusion strategies can be categorized as feature-level, intermediate, or decision-level. Feature-level fusion concatenates modality-specific representations, whereas intermediate fusion aligns latent spaces through joint embedding or attention mechanisms. Decision-level fusion aggregates independent predictions using weighted or probabilistic schemes.

Future radiomics research should favor fusion strategies that explicitly model modality uncertainty and conditional dependence. Naive concatenation amplifies noise and correlation effects. Modality-aware weighting, attention-based fusion, and Bayesian integration offer more principled alternatives, particularly in settings with missing or partially observed modalities.

### 10.4. Self-Supervised and Representation Learning

Self-supervised learning provides a mechanism for representation learning without manual labels. Common objectives include contrastive learning, reconstruction-based learning, and predictive pretext tasks. In radiomics, self-supervised pretraining can be applied at the image or region level to improve feature robustness prior to downstream modeling [82].

Integration of self-supervised representations with handcrafted radiomic features raises several technical questions, including representation alignment, redundancy control, and interpretability preservation. Empirical evaluation should assess whether self-supervised features improve generalization under domain shift and whether they maintain stability under acquisition variability.

### 10.5. Federated Radiomics

Federated learning enables distributed model training across institutions without centralized data aggregation [83]. In radiomics, federated settings introduce non-identically distributed data due to scanner, protocol, and population differences. These factors complicate optimization and convergence.

Technical challenges include client drift, communication efficiency, and aggregation bias. Methods such as weighted aggregation, domain-aware optimization, and federated feature normalization require further investigation. Evaluation of federated radiomics models should include cross-site generalization and stability analysis rather than aggregated performance alone.

### 10.6. Integration of Radiomics into Clinical PACS Workflows

Integration of radiomics software into Picture Archiving and Communication System (PACS) environments represents an important step toward routine clinical adoption. Embedding radiomics pipelines directly within radiology workflows could enable automated feature extraction, real-time decision support, longitudinal imaging analysis, and seamless interaction with electronic health record systems. Such integration may reduce barriers between research and clinical implementation by improving usability, workflow efficiency, and accessibility for radiologists. However, practical deployment requires standardized imaging protocols, interoperable software frameworks, regulatory compliance, computational scalability, and transparent reporting of radiomic features and model outputs. Future radiomics systems should therefore emphasize clinically interpretable and workflow-compatible implementations rather than isolated research-only frameworks.

### 10.7. Standard Benchmarks and Reporting Standards

Radiomics lacks standardized benchmarks that support reproducible method comparison. Existing studies vary widely in task definition, cohort composition, preprocessing, and validation design. This heterogeneity limits cross-study comparison and cumulative evidence synthesis.

Future benchmarks should define fixed training–validation–test splits, standardized preprocessing pipelines, and reference evaluation metrics. Reporting standards should mandate disclosure of feature definitions, discretization parameters, validation nesting, and uncertainty estimates. Without such standardization, performance comparisons remain inconclusive and clinically uninformative.

## 11. Conclusions

Radiomics provides a structured framework for quantitative medical image analysis, offering interpretable feature representations that remain effective in limited-data clinical settings. This survey presented an end-to-end, methodology-centric analysis of radiomics pipelines, emphasizing how design choices across acquisition, preprocessing, segmentation, feature engineering, modeling, and validation jointly determine reproducibility, robustness, and translational validity.

Despite sustained progress, radiomics is constrained by feature instability, sensitivity to pipeline variability, high dimensionality under limited sample sizes, and persistent validation bias. Inconsistent standardization and limited external evaluation further restrict generalization and clinical adoption. These limitations underscore that reported performance gains often reflect methodological artifacts rather than robust predictive capability.

This review is limited by its focus on methodological analysis rather than quantitative performance aggregation, as heterogeneity across datasets, tasks, and validation protocols precludes meaningful meta-analysis. Coverage reflects representative trends in the literature rather than exhaustive benchmarking or disease-specific optimization.

Future progress depends on standardized pipelines, stability-aware feature selection, leakage-free validation, and principled integration with DL and multimodal frameworks. Radiomics remains a valuable but fragile paradigm; its long-term impact will be determined by methodological rigor rather than increasing model complexity alone.

## Figures and Tables

**Table 1:** Summary of review methodology components and criteria.

Component	Description
Literature Sources	IEEE Xplore, PubMed, Scopus, Web of Science, Springer, Elsevier, and Google Scholar; publications up to 2026.
Study Types	Peer-reviewed journal articles, conference proceedings, and selected high-impact preprints with methodological contributions.
Inclusion Criteria	Studies involving quantitative radiomic feature extraction from medical images (CT, MRI, PET, ultrasound); explicit discussion of feature engineering, preprocessing, segmentation, feature selection, dimensionality reduction, modeling, or validation protocols.
Exclusion Criteria	End-to-end Deep Learning (DL) studies without explicit radiomic feature analysis; application-only studies lacking methodological detail; non-medical imaging domains; editorials, abstracts without full manuscripts, and non-English works.
Screening Process	Two-stage screening comprising title/abstract filtering followed by full-text review. Ambiguous cases were resolved based on relevance to pipeline design, reproducibility, and validation methodology.
Extracted Attributes	Imaging modality, preprocessing and harmonization methods, segmentation strategies, feature categories, feature selection and dimensionality reduction techniques, modeling approaches, validation protocols, and reported limitations (e.g., instability, leakage, generalization gaps).
Synthesis Approach	Qualitative comparative synthesis emphasizing methodological dependencies, feature stability, reproducibility, and validation practices rather than task-specific performance metrics.

**Table 2:** Acquisition-related sources of variability, affected radiomic feature categories, and commonly adopted mitigation strategies.

Variability Source	Affected Features	Mitigation Strategy
Slice thickness/resolution	Texture, wavelet	Isotropic resampling
Reconstruction kernel	Texture, higher-order	Kernel harmonization
Scanner manufacturer	Most feature categories	ComBat harmonization
Intensity scaling	First-order, texture	Intensity normalization

**Table 3:** Taxonomy of radiomic feature categories and their key characteristics.

Feature Category	Mathematical Basis	Stability	Interpretability	Sensitivity
First-order	Intensity statistics	High	High	High
Shape	Geometric descriptors	High	High	High
Texture	Spatial dependency matrices	Moderate	Moderate	High
Higher-order	Filtered or transformed features	Low–Moderate	Low	High

**Table 4:** Comparison of commonly used radiomics software frameworks and their methodological characteristics.

Framework	Implementation	Feature Support	IBSI Compliance	Key Characteristics
PyRadiomics	Python (v3.0.1)	First-order, shape, texture, higher-order	Yes	Open-source, reproducible, widely adopted in ML workflows
IBEX	MATLAB (R2023a)	Texture and quantitative imaging biomarkers	Partial	Supports visualization and feature analysis
MaZda	Standalone software (v4.6)	Statistical and texture features	Limited	Early radiomics framework with handcrafted texture analysis
CERR	MATLAB (R2023a)	Radiomics and radiotherapy imaging features	Partial	Supports multimodal imaging and radiotherapy integration

**Table 5:** Methodological factors affecting stability, reproducibility, and clinical validity in radiomics.

Pipeline Component	Methodological Choice	Effect on Stability	Reproducibility Concern	Clinical Implication
Image acquisition	Heterogeneous scanners and protocols	Alters intensity and texture features	Reduced cross-site consistency	Limited generalization
Preprocessing	Normalization and resampling	Stabilizes feature distributions	Preprocessing-induced feature drift	Reduced deployment reliability
Gray-level discretization	Fixed bin width/count	Affects texture computation	Inconsistent feature extraction	Poor biomarker reproducibility
Segmentation	Manual ROI delineation	Boundary-sensitive features	Observer variability	Limited clinical consistency
Automated segmentation	DL-based segmentation	Improved consistency	Domain shift sensitivity	Requires external validation
Feature extraction	First-order and shape features	Relatively stable descriptors	Acquisition sensitivity	Better interpretability
Texture features	GLCM, GLRLM, GLSZM	Noise-sensitive descriptors	Low preprocessing robustness	Unstable signatures
Higher-order features	Wavelet and filtered features	High preprocessing sensitivity	Increased inter-study variability	Reduced multicenter reliability
Feature selection	LASSO, mRMR, RFE	Removes unstable features	Selection variability across folds	Affects model robustness
Dimensionality reduction	PCA and latent projection	Compresses feature space	Leakage risk before splitting	Optimistic performance bias
Model development	Ensemble and DL models	Increased predictive capacity	Overfitting in small cohorts	Reduced interpretability
Validation strategy	Internal validation only	Optimistic estimates	Limited generalizability	Weak clinical confidence
External validation	Multi-center evaluation	Tests domain robustness	Improved reproducibility assessment	Better translational reliability
Harmonization	ComBat and feature harmonization	Reduces scanner variability	Additional modeling assumptions	Improved multicenter applicability
Leakage prevention	Fold-wise preprocessing	Preserves unbiased estimation	Prevents inflated performance	Reliable clinical evaluation

**Table 6:** Feature selection strategies in radiomics and their methodological trade-offs.

Method	Robustness	Leakage Risk	Interpretability
Filter	Low–Moderate	Moderate	High
Wrapper	Low	High	Moderate
Embedded	Moderate	Moderate	High
Dimensionality reduction	Moderate	Moderate	Low

**Table 7:** Comparison of radiomics and DL approaches in medical image analysis.

Features	Radiomics	DL
Data and annotation requirements	Effective with small cohorts; relies on accurate ROI delineation	Requires large, well-annotated datasets
Feature representation	Handcrafted, predefined, interpretable features	Automatically learned hierarchical representations
Interpretability	High feature-level transparency	Limited; relies on post-hoc explainability methods
Computational demands	Moderate; feasible on standard infrastructure	High; typically requires GPUs and extensive training
Robustness and generalization	Sensitive to acquisition variability; mitigated via harmonization	Sensitive to dataset bias; improves with data diversity and augmentation
Reproducibility and validation	Affected by preprocessing and feature stability; requires external validation	Affected by architectural and training stochasticity; requires large validation cohorts
Clinical translation	Higher acceptance and easier deployment	Slower adoption due to trust and infrastructure requirements
Representative use cases	Biomarker discovery, prognostic modeling, low-data studies	Detection, segmentation, large-scale prediction tasks

**Table 8:** Classical radiomics-based machine learning studies for tumor characterization and classification between 2017–2019.

Study (Year)	Modality and Clinical Task	Feature and Model	Key Limitations
[47] Yu et al. (2017)	CT; differentiation of renal tumor subtypes and oncocytoma	Histogram, texture, gradient features; linear SVM	Handcrafted features only; limited robustness to segmentation variability; single classifier
[48] Lu et al. (2018)	MRI; glioma molecular subtype stratification	Intensity, texture, shape features; ML classifiers (SVM)	High-dimensional handcrafted features; limited generalization across scanners and protocols
[49] Feng et al. (2018)	CT; differentiation of renal mass types	Texture features; SVM with RFE and SMOTE	Synthetic oversampling introduces bias; manual ROI delineation
[51] Chaddad et al. (2019)	MRI; survival prediction in recurrent glioblastoma	CNN-based deep radiomic features; RF	Limited interpretability; data-intensive deep features
[52] Li et al. (2019)	PET/CT; survival prediction in rectal cancer	Deep CNN with spatial pyramid pooling	Requires large datasets; limited explainability
[53] Chen et al. (2019)	MRI; glioblastoma vs. metastatic brain tumors	Texture features; distance correlation feature selection with LDA/LR	Sensitivity to feature selection strategy; handcrafted features

**Table 9:** Classical radiomics-based machine learning studies for tumor characterization and classification beyond 2020.

Study (Year)	Modality and Clinical Task	Feature and Model	Key Limitations
[54] Yi et al. (2020)	CT; grading of clear cell RCC (low vs. high grade)	Radiomic features; SVM and RF	Binary grading task only; limited subtype coverage
[55] Gitto et al. (2020)	MRI; grading of cartilaginous bone tumors	First-order and texture features; AdaBoost	Limited evaluation across tumor types; potential overfitting
[56] Deng et al. (2020)	CT; benign vs. malignant renal tumor differentiation	Filtration–histogram texture features; LR	ROI drawn on single slice; limited 3D tumor representation
[57] Erdim et al. (2020)	CT; benign vs. malignant renal tumors	Texture features; DT, k-NN, LR, SVM, NB, RF, FFN	Extensive model comparison without unified optimization; handcrafted features
[58] Nie et al. (2020)	CT; fat-poor angiomyolipoma vs. ccRCC	Radiomics signature with clinical factors; nomogram	Limited external validation; dependence on clinical variables
[59] Sun et al. (2020)	CT; benign vs. malignant renal tumors	Qualitative radiologic + quantitative texture features; SVM	Manual feature design; limited scalability
[60] Uhlig et al. (2020)	CT; five renal tumor subtype classification	Radiomic features; XGBoost and LR with RFE	Feature pruning sensitive to training data; class imbalance
[61] Wang et al. (2021)	CT; ccRCC vs. non-ccRCC classification	Radiomic features; RF, SVM, LR	Limited interpretability of ensemble models; correlation-based feature removal
[62] Gurbani et al. (2021)	CT (non-contrast and portal venous); aggressive RCC phenotype prediction	3D volumetric radiomic features; XGBoost, RF, SVM	Focus on large tumors only; complex feature selection pipeline

**Table 10:** A comparison of deep radiomics and hybrid radiomics–DL frameworks.

Study (Year)	Modality and Clinical Task	Feature and Model	Key Limitations
[63] Alhussaini et al. (2022)	CT; malignant renal tumor differentiation	Handcrafted radiomic features; sparsity-driven feature reduction with RF, SVM, k-NN, LR, NB	Limited feature diversity; reliance on handcrafted descriptors
[65] He et al. (2023)	CT; malignancy risk prediction in cystic renal lesions	Handcrafted radiomics + DL features from pretrained ResNet; GB, XGBoost, DT	Increased model complexity; feature fusion strategy not fully interpretable
[67] Xu et al. (2023)	CT; binary renal tumor classification	Radiomics + clinical attributes with RF, XGBoost, and CNN	Heterogeneous data integration; potential clinical data dependency
[70] Kilicarslan et al. (2025)	MRI; RCC subtype classification	Transfer learning with pretrained DenseNet, GAP-based deep features; SVM	Dependence on pretrained models; limited interpretability of deep features
[64] Lam et al. (2022)	MRI; tumor mutational burden prediction in lower-grade gliomas	Handcrafted radiomic features; LightGBM	Limited biological interpretability; scanner variability sensitivity
[66] Kumar et al. (2023)	MRI; low- vs. high-grade glioma classification	Radiomic features; SVM, RF, GB, NB, AdaBoost	Handcrafted features only; binary grading task
[68] Magnuska et al. (2024)	Ultrasound; binary breast tumor classification	Radiomics + DL features; SVM, RF, LR	Operator-dependent US acquisition; limited generalization
[69] Chaddad et al. (2025)	MRI; glioma grading and survival prediction	Radiomic features with selection; XGBoost, RF	Feature-selection sensitivity; survival modeling complexity

## Data Availability

The original contributions presented in this study are included in the article. Further inquiries can be directed to the corresponding author.
